# Phylo-evo-devo: combining phylogenetics with evolutionary developmental biology

**DOI:** 10.1186/1741-7007-7-36

**Published:** 2009-06-26

**Authors:** Alessandro Minelli

**Affiliations:** 1 Department of Biology, University of Padova, I 35131 Padova, Italy

## Abstract

As a result of the integration of molecular and morphological approaches for the reconstruction of phylogenies, and of the intertwining of developmental and evolutionary biology, further prospects are open for a fruitful interaction between these two fields in what we may call a phylo-evo-devo approach.

Wiegmann et al.'s molecular phylogeny of the holometabolous insect orders, recently published in BMC Biology, offers a good opportunity to revisit the inverted positions of wings and halteres in the Diptera and the Strepsiptera in terms of a putative homeotic mutation in the Hox gene Ultrabithorax. The main finding of this paper is that Strepsiptera are closely related to the Coleoptera rather than Diptera, as recently claimed. Through this exemplary case, the paper demonstrates the value of the reciprocal illumination we can expect from the integration of a good phylogeny and a sound knowledge of the evolvability of developmental mechanisms.

## Commentary

Evolutionary developmental biology, or evo-devo, has benefited from an integrated use of tools, both conceptual and experimental, that were originally developed within a number of separate fieldsand the discipline'smultiple roots are fixed inits name. Another discipline to benefit is phylogenetics, within which the morphological and the molecular approach are far from being quietly integrated, but are nevertheless in daily, eventually constructive confrontation. Needless to say, an integration of evo-devo with phylogenetics, an approach we may call phylo-evo-devo, will benefit both traditions alike, the phylogenetic and the evolutionary developmental one [1]. What matters, however, is to take the new challenge seriously. If evo-devoists have been too often naïf in reconstructing ancestral states without resorting to cladistics, phylogeneticists have been equally naïf in using vague notions of comparative developmental genetics to speculate too freely about the possible origin, or course, of major morphological changes in evolution.

Evo-devo was called in as a possible source of explanation of the counterintuitive morphological relationships between two insect orders, the Diptera and the Strepsiptera, whose close phylogenetic affinities were suggested by mitochondrial DNA sequences [2-7]. In male Strepsiptera (females are wingless) the forewings are reduced to small rigid structures somehow comparable to the modified hindwings (the halteres) of the Diptera, while their hindwings are membranous (as are the hindwings of the Diptera). Morphologists had never suggested dipteran affinities for the Strepsiptera, the most popular phylogenetic hypothesis favouring instead affinities to the Coleoptera, with the Hymenoptera being sometimes offered as an alternative. Now, under the new hypothesis of sister-group relationships between the Diptera and the Strepsiptera, the rough similarity between the dipteran halteres and the reduced forewings of the Strepsiptera was deemed worthy of investigation. The comparison, however, stood against a great difficulty: the lack of positional equivalence between the two structures. Thus, knowing that in the fruitfly the identity of the dorsal appendages of the meso- and metathorax is under the control of the Hox genes, it was suggested that the inverted position of membranous wings and halteres between the two insect orders could be the result of a point mutation of one of these genes (tentatively, Ultrabithorax) in a common ancestor with dipteran-like organization [2]. This hypothesis, however, disregarded the fact that all known mutations in Hox genes eventually resulting in the positional shift of a given pair of appendages are either a gain-of-function or a loss-of-function mutation, respectively producing, at a given position along the main body axis, a feature originally expressed at a more anterior or posterior location. No mutation, however, is known to cause a reciprocal exchange of position between two features, as required by the 'homeotic wing/haltere exchange' hypothesis. The problem thus remained unresolved, as further phylogenetic analyses based on mitochondrial gene sequences seemed to confirm the dipteran affinities of the Strepsiptera.

The problem was clearly in need of further study. At last, this has been done in a newly published analysis of the phylogenetic interrelationships among all the orders of holometabolous insects [8]. This study, based on a reasonably rich sample of nuclear gene sequences, rejects the putative affinities of the Strepsiptera with the Diptera and restores ample credibility to the old hypothesis of closest affinities between the Strepsiptera and the Coleoptera. Thus, if any morphological comparison is eventually worth investigation among the dorsal thoracic appendages of these insects, the scenario must now involve the 'halteres' of male Strepsiptera and the elytra of the Coleoptera – obvious positional homologues, as they all belong to the second thoracic segment.

Morphological evidence is probably insufficient to reconstruct phylogeny whenever structures are extensively reduced [9], as in the case of the forewings of the Strepsiptera, or the halteres of the Diptera. In these cases, to rely more on molecular than morphological evidence is an obvious choice, but this does not mean that morphology cannot be informative. This is particularly true of those instances where evolutionary developmental biology is able to throw light on changes in morphology. Vertebrates with reduced limbs are a case in point, as the order with which the digits are lost, in the species with less than five, turns out to be clade-specific and to strictly correspond, in inverted order, to the sequence with which the five digits are built in those related species that have all of them [10]. As a consequence, the simple inspection of the hand or the foot is sometimes of value in assessing the affinities of animals with two, three or four digits, and this is obviously relevant when we deal with fossils.

There are instances where phylogeny helps to restrict the choice among alternative hypotheses of developmental mechanisms responsible for producing a given structural trait. This is the case of arthropod segmentation. Two competing scenarios have been proposed, one of them implying that segmental units are produced serially by the activity of a posterior, subterminal growth zone. The other scenario suggesting instead that an initial establishment of a small number of primary segmental units along the anterior-posterior body axis is followed by the regular splitting of these units into secondary segments, through one or more runs of duplication. Of recent, a scolopender species has been described, with leg-bearing segments numbering either 39 or 43 within the same population, thus dramatically differing from all the remaining 700 species in that group, which have either 21 or 23 pairs of legs. Interestingly, a cladistic analysis has shown that this centipede does not represent an isolated lineage, but has recently split off from within a genus where the more popular leg-pair numbers 21 and 23 can coexist in the same species. The total lack of intermediates and the roughly 2:1 ratio in segment number between the new species and its relatives seems to be a good argument in favour of the double-segmentation model [11].

We now have more and more robust phylogenies and deeper insights into evolutionary variations of developmental mechanisms, but the challenge is to understand the data in an integrated phylo-evo-devo framework. Further levels of integration are waiting around the corner. For example, evo-devo has being largely growing on the recent spectacular results of comparative developmental genetics, but this still requires a good injection of population genetics [12]. A few pilot studies in this direction [13,14] have amply demonstrated how much this helps the understanding of evolvability, arguably one of the most exclusive topics of evo-devo research [15].

## About the author

Alessandro Minelli is Professor of Zoology at the University of Padova. Following a long interest in myriapod systematics and evolution, he is currently active in evolutionary developmental biology, with a focus on segmentation and the evolution of the life cycle.
